# Immunoexpression of Wnt/β-catenin signaling pathway proteins in ameloblastoma and calcifying cystic odontogenic tumor

**DOI:** 10.4317/jced.53100

**Published:** 2017-01-01

**Authors:** Sabrina-Nogueira Dutra, Fábio-Ramôa Pires, Luciana Armada, Rebeca-Souza Azevedo

**Affiliations:** 1 PhD, Oral Pathology, Piracicaba Dental School, State University of Campinas, Piracicaba/SP, Brazil; 2 Professor, Oral Pathology, School of Dentistry, State University of Rio de Janeiro, Brazil; Professor, Post-graduate program in Dentistry, Estácio de Sá University, Rio de Janeiro/RJ, Brazil; 3 Professor, Post-graduate program in Dentistry, Estácio de Sá University, Rio de Janeiro/RJ, Brazil; 4 Professor, Patologia Oral, Faculdade de Odontologia, Universidade Federal Fluminense, Nova Friburgo, Rio de Janeiro/RJ, Brazil

## Abstract

**Background:**

Wnt/β-catenin signaling pathway is essential for the beginning of odontogenesis and may be involved in the development and progression of some odontogenic tumors. Thus, the aim of this study was to comparatively evaluate the immunohistochemical expression of Wnt/β-catenin signaling pathway proteins in a series of AME and CCOT.

**Material and Methods:**

Immunohistochemical reactions were performed using antibodies against Wnt1, Wnt5a and β-catenin in 17 cases of solid AME and 6 cases of CCOT.

**Results:**

In the AME group, Wnt1 and Wnt5a were identified in the epithelium in most of the cases, and β-catenin was mainly identified in the cytoplasm of the tumoral cells. In the CCOT group, Wnt1 and Wnt5a were identified in the epithelium and in the ghost cells in almost all the cases, and β-catenin was mainly identified in the cytoplasm and in the nuclei of the tumoral cells.

**Conclusions:**

These results contribute to support the importance of Wnt/β-catenin signaling pathway proteins in AME and CCOT tumorigenesis. The abnormal expression of cytoplasmic and/or nuclear β-catenin appears to contribute to the development of both AME and CCOT. In addition, it is possible that Wnt1 and Wnt5a expression in ghost cells can contribute to its histogenesis in CCOT.

** Key words:**Ameloblastoma, β-catenin, calcifying cystic odontogenic tumor, immunohistochemistry, Wnt.

## Introduction

The Wingless type (Wnt)/β-catenin signaling pathway is essential for early odontogenesis activation (1). There is also additional evidence of the involvement of this pathway in the development of some odontogenic tumors (OT), such as ameloblastoma (AME) (2-4) and calcifying cystic odontogenic tumor (CCOT) (4-6). Classical Wnt/β-catenin signaling pathway, also named the canonical pathway, is usually activated by Wnt1 protein and inactivated by Wnt5a protein (7). The activation of this pathway will stabilize β-catenin, allowing its cytoplasmic accumulation and nuclear translocation; in the nucleus, β-catenin will participate in the expression of genes related to the cell cycle during embryogenesis and during the development of some benign and malignant tumors (7-9).

AME and CCOT figure among the four more common OT, comprising, respectively, around 30% and 7% of all OT in a recent series reported (10). AME is considered a locally aggressive benign OT classified in four different clinicopathologic variants: solid, unicystic, peripheral and desmoplastic (11,12). CCOT is a benign cystic OT that is microscopically characterized by an ameloblastoma-like lining epithelium associated with the presence of ghost cells (13), and may be associated with an ameloblastomatous proliferation in some cases (14).

Herein, the aim of this study was to evaluate the immunohistochemical expression of Wnt/β-catenin signaling pathway proteins in AME and CCOT in order to highlight the involvement of this pathway in their development.

## Material and Methods

A total of 17 solid AME and 6 CCOT were selected from the files of the Oral Pathology Laboratory, School of Dentistry, State University of Rio de Janeiro, Brazil.

**Figure 1:**
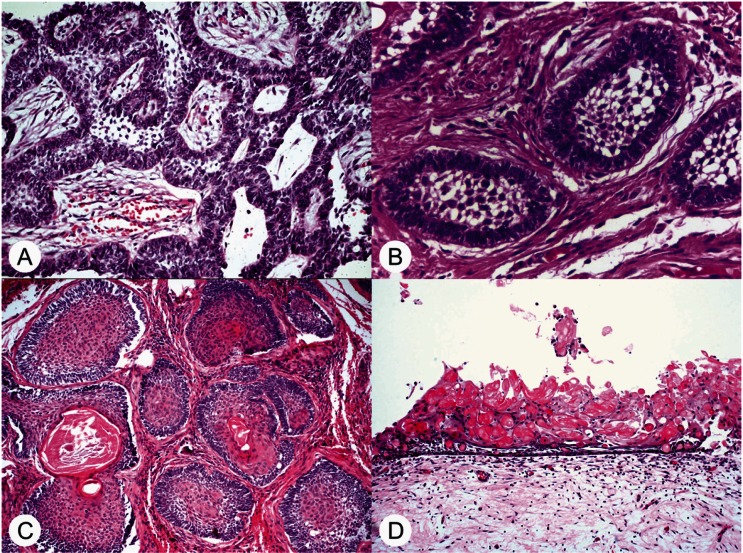
Histological subtypes of AME and CCOT included in this study (hematoxylin and eosin). A, AME plexiform (original magnification, x200); B, AME follicular (original magnification, x200); C, AME acanthomatous (original magnification, x200); and D, CCOT showing a cystic cavity lined by an ameloblastomatous-like epithelium containing a great amount of ghost cells (hematoxylin and eosin, original magnification, x100)

AME affected 10 males and 7 females with ages ranging from 12 to 72 years (mean of 33.9 years). Mandible was affected in 14 cases. All cases were microscopically reviewed and classified according to the histological classification of AME described by the World Health Organization Histological Classification of Odontogenic Tumors (11). Seven cases were classified as plexiform, 4 cases as follicular, 1 case as acanthomatous and in 5 cases there was an hybrid pattern including more than one histological sub-type (Fig. 1A-C).

CCOT affected 6 females with ages ranging from 10 to 28 years (mean of 17 years). Mandible and maxilla were affected in 3 cases each. None of the 6 CCOT were associated with any other OT. All cases were microscopically reviewed and classified according to the histological classification of CCOT (15) (Fig. 1D).

For the immunohistochemical reactions, 3-μm tissue sections on silane-coated histological slides were incubated with primary antibodies against Wnt1 (polyclonal; R&D Systems; 1:100), Wnt5a (clone 442625; R&D Systems; 1:50) and β-catenin (clone 17 C 2; Novocastra; 1:100) by using the labeled streptavidin-biotin kit (LSAB, Dako) using the immunoperoxidase technique. Visualization was acquired with 3,3’diaminobenzidine solution (DAB, Dako) and Carazzi’s hematoxylin counterstaining. Positive controls were tumor cells from a breast carcinoma (Wnt1), small intestine’s villus (Wnt5a) and the lining epithelium of a fibrous hyperplasia (β-catenin). Internal adjacent tissues of each positive control was used as a negative reference of the immunohisto-chemical reactions. Wnt1 and Wnt5a expression was scored as undetectable or detectable, if >5% of tumor cells cytoplasm were positive, and β-catenin expression was scored as negative, positive on cell membrane, positive on cell membrane and cytoplasm, positive on cytoplasm, or positive on cytoplasm and nucleus. These immunohistochemical expressions were descriptively presented.

This study was carried out with the approval of Research Ethics Committee (016/2011, May 9, 2011, School of Dentistry of Piracicaba, State University of Campinas, Brazil) and financial supported by Fundação Carlos Chagas Filho de Amparo à Pesquisa do Estado do Rio de Janeiro (FAPERJ) and Coordenação de Aperfeiçoamento de Pessoal de Nível Superior (CAPES).

## Results

**Figure 2:**
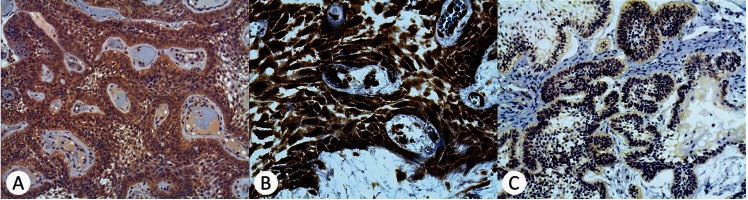
Immunoexpression of Wnt/β-catenin signaling pathway proteins in AME epithelium (immunoperoxidase). A, Wnt1 identified in a plexiform AME (original magnification, x200); B, Wnt5a identified in a plexiform AME (original magnification, x400); C, β-catenin identified in a follicular AME (original magnification, x100).

Wnt1 was detectable in the epithelium from 12 cases of AME (64.5%) and Wnt5a was detectable in the epithelium from 9 cases of AME (53%). β-catenin showed cytoplasmic staining in 12 cases (70.6%), cytoplasmic and membranous staining in 2 cases (11.8%) and was undetectable in the epithelium from 3 cases of AME (17.6%) (Fig. 2).

**Figure 3:**
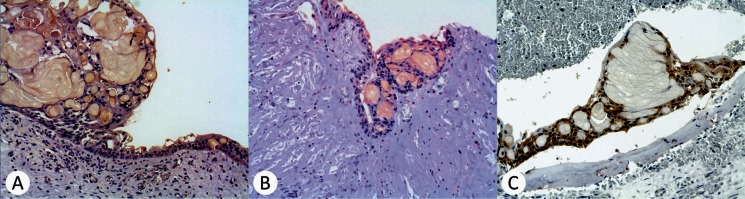
Immunoexpression of Wnt/β-catenin signaling pathway proteins in CCOT ameloblastoma-like epithelium and ghost cells (immunoperoxidase). A, Wnt1 identified in the epithelium and ghost cells of a CCOT (original magnification, x200); B, Wnt5a identified in the epithelium and ghost cells of a CCOT (original magnification, x400); C, β-catenin identified only in the epithelium of a CCOT (original magnification, x100).

Wnt1 and Wnt5a were heterogeneously detectable in the lining epithelium from all cases of CCOT (100%). Wnt1 was also detectable in the ghost cells from 5 CCOT (83.3%) and Wnt5a was detectable in the ghost cells from all CCOT (100%). β-catenin showed both cytoplasmic and nuclei staining in the lining epithelium from 5 CCOT (83.3%) and was undetectable in the ghost cells from all CCOT (100%) (Fig. 3).

## Discussion

Wnt family of glycoproteins is one of the major families of signaling molecules expressed during the development of a multicellular organism and, in adult tissues; they may be expressed in homeostatic renewal or in tumorigenesis of some benign and malignant neoplasms (9,16,17). Traditionally, members of this family are divided into two groups according to the potential of activating the classical canonical Wnt/β-catenin signaling pathway. The Wnt1 class has a greater potential for activating the canonical pathway than the Wnt5a class (7,18). Furthermore, it is known that Wnt5a may also act as an antagonist of this pathway (18,19).

Wnt1 expression had already been demonstrated in AME (16,20,21). It is usually the most common Wnt family glycoprotein expressed in AME epithelium, regardless of the clinicopathologic subtype (16,20). Nevertheless, a study had not identified Wnt1 positivity in follicular AME (21). Wnt1 overexpression in the present series of AME is justified since Wnt1 is considered the most potent canonical signaling pathway activator (7) and, as a consequence of this pathway activation, a high cytoplasmic expression of β-catenin was also identified (8). Even so, the expected nuclear β-catenin translocation was not identified in the AME epithelium from our cases. It is noteworthy that an overexpression of Wnt1 was similarly identified in keratocystic odontogenic tumor, a common and benign OT that may also have an aggressive behavior (22).

Wnt5a function is conflicting since it may activate or inactivate the canonical Wnt/β catenin signaling pathway (7,16,17). Likewise, it is interesting to notice this Wnt5a antagonism in the literature, as its expression can vary from 0 to 100% in different series of AME (4,16,23). This great variation seems to be dependent on differentiation of tumor cells or on cell surface receptors available, or by the use of different clones of the antibody (4,16,19). In the present series of AME, Wnt5a was identified in about half of the cases, which may be justified by the possibility of this glycoprotein activate or inactivate the canonical Wnt/β catenin signaling pathway and by the fact that AME also present a great variation of radiographic presentation including from minor to major lesions (12,16).

Wnt1 and Wnt5a immunoexpression on CCOT revealed to be heterogeneous and consistent positive on the lining epithelium of this OT. Considering the benign and non-aggressive clinical behavior of CCOT, we could speculate that Wnt5a acts as an inhibitor of canonical Wnt/β catenin signaling pathway in balance with the Wnt1 activator. To support this statement, it is noteworthy that evidences of the suppressor potential of Wnt5a had been already described in the literature in AME, a benign locally aggressive OT (16). Also, Wnt1 and Wnt5a positivity was identified in the ghost cells of CCOT in the present study, a finding not previously reported. Once the canonical Wnt/β catenin signaling pathway is probably activate, the expected β catenin cytoplasm and/or nucleus expression in the ghost cells of the present series of CCOT was not identified. β catenin had neither been identified in the ghost cells in CCOT, pilomatrixoma and craniopharyngioma in a study concerning the main entities microscopically characterized by the presence of ghost cells (24), but it was previously identified on ghost cells cytoplasm from odontomas (25). Thus, in brief, it seems that only Wnt plays a role in both CCOT and ghost cells histogenesis (26).

β-catenin accumulation in the cytoplasm will allow β-catenin translocation into the nucleus, and consequently, will induce the expression of different genes associated with cell proliferation, as already demonstrated in odontogenesis and in some benign and malignant neoplasms (6-8,27). It is interesting to notice that even when cytoplasmic and nuclear β-catenin accumulation have been identified in AME (3,8,28), a mutation in the gene encoding this protein was not identified (2,8,29,30). Unlikely, this muta-tion had already been identified in CCOT (2,6), which also were immunohistochemically positive for cytoplasmic and nuclear β-catenin in the ameloblastoma-like epithelium of most of the cases (5,6,25), as seen in the present study.

Additionally, we were not able to observe a single AME presenting nuclear β-catenin immunoexpression in the present series, which may be a result of the limited number of cases. Another interpretation is that the common presence of Wnt5a, a glycoprotein capable of inhibiting the Wnt/β catenin signaling pathway, could promote this low β-catenin nucleus accumulation. Nevertheless, the unexpected β-catenin cytoplasm accumulation is an important pre-requisite for β-catenin nucleus accumulation and it was observed in nearly 80% of the cases of AME.

The present study contributes to reinforce the possible relationship of the canonical Wnt/β-catenin signaling pathway in the process of tumorigenesis in both AME and CCOT, especially because the positive relationship between Wnt1 immunoexpression and cytoplasmic and/or nuclear β-catenin immunoexpression in the epithelium of both AME and CCOT. Wnt5a immunoexpression identified in half of the cases of AME may express both its activating and inactivating role; and Wnt5a immunoexpression identified in all cases of CCOT may express a balance between the activator Wnt1 and the inactivator Wnt5a in CCOT. It also seems that Wnt1 and Wnt5a take part of ghost cells development in CCOT. Further studies concerning these and other components of this signaling pathway, including the use of techniques that demonstrate the activity of these molecules, may contribute to elucidate the pathogenesis of both AME and CCOT.
